# “Medical Education” and Our Colleges as Contributors Thereto

**Published:** 1884-08

**Authors:** P. P. Wells


### HOMŒOPATHIC PHYSICIAN.

A MONTHLY JOURNAL OF MEDICAL SCIENCE.

"If our school ever gives up the strict inductive method of Hahnemann, we are lost, and deserve only to be mentioned as a caricature in the history of medicine."—CONSTANTINE HERING.

Vol. IV. AUGUST, 1884. No. 8.

### "MEDICAL EDUCATION" AND OUR COLLEGES AS CONTRIBUTORS THERETO.

Medical education has become a standard topic for consideration in all our largest gatherings of doctors, in institutes, associations, societies—large and small; and a very important topic it is and has been for no little time. Much has been said and written on the subject. Each year and each meeting of these bodies brings to our knowledge that there are minds very conscious of a want of something better in this than is found in our present experience, and these repeat each year and in each meeting the utterances of previous years and meetings, from which has come, as yet, small fruits of increase of knowledge, which alone may be supposed to constitute the something better so earnestly desired and advocated. Repetitions of these commonplace utterances, so cheap and so often heard, have added to no man's knowledge of the ways and means of healing the sicknesses of mankind, which we understand to be the only legitimate objective of "medical education." That process or those teachers which fail to give this knowledge to medical pupils have done *nothing* toward imparting to them a *true* medical education, and it or they, who only repeat this year the work of the last and preceding years, have done nothing toward securing that "better" so long talked of and apparently so earnestly desired. Why should so good a work *stop*, so just *before* it begins?

The first and most obvious reason for this is a want of clear perception by these talkers of what constitutes a *true* medical education, and none whatever of that which would go to make up the "better," so sorely needed, as an addition to present attainments, or of how this is to be gained. Medical education in general may be regarded as embracing a knowledge of practical medicine—*i. e.*, healing the sick by the use of medicines—and practical surgery—*i. e.*, healing such diseases and results of mechanical injuries as require mechanical means for their cure more or less, the two branches being included in the curriculum the neophyte is to pass before he is turned out of his college into the world to do his best for the sick or maimed who may trust him with the duty of their healing. So it will be seen his education is supposed to have given him a knowledge of many sciences, which, together, make up the idea of the education the healer should have received, and this wholly irrespective of the literary and art culture needful to prepare him to receive a knowledge of these sciences most perfectly. Such of these sciences as comprise the materialism of their professional education may be supposed to have been measurably well taught and acquired by the learner, viz.: Anatomy, physiology, pathology, pathological anatomy, histology, etiology, semeiology, diagnosis, prognosis, chemistry, and physics. He may go out for the duties of his practical life with a respectable knowledge of all these, or even he may be a master of them all (a rarity), and yet before sicknesses he may be called to cure, he will sigh for a something more and "better" than these; for neither of these nor all of them combined, however valuable as constituents of necessary knowledge to the healer, have any word as to the particular agent required for the cure of the case before him, and the knowledge of this is just what he wants now for relief of his patient and his own anxiety. It is not in these sciences which he may have been well taught, and may know with passable thoroughness, that that knowledge is found which equips him as a healer and gives him, before sicknesses with which he has to deal, the comfortable assurance that he is their master. With whatever of mastery of these sciences he may have, in spite of them all he stands before sickness powerless for its cure unless he has added to these the knowledge which enables him to lay his hand on the specific curative for the case before him. Without this knowledge he is in all clinical duties a doomed guesser and blunderer for life.

It is not, then, in these branches of professional knowledge that the sighed for and needed "better" is to be sought or found. Then, it may be pertinently asked, why is it that the best talent and most earnest labors of our schools are spent on these, while the knowledge which enables one to *cure* is comparatively so greatly neglected?* And certain it is, that if any improvement is to be realized in our medical education, it is just here it must be found. Not that these named branches of knowledge are to be less perfectly or less earnestly taught, but that this other and more important than them all—the science of therapeutics—is to be less neglected. The other sciences named are of no practical value to the healer except as aids to the right administration of this last. Then, we repeat, why are these so sedulously cultivated and this so grossly neglected? Is it not apparent as the light of day that the "improvement" in medical education so much talked of and so greatly needed is in the reversal of this practice in our schools?—not in any neglect of these aids, but in giving proper and greater endeavor to this principal science, in which only is true philosophy of diseases and their curatives, and which discloses their relationship and teaches how the agent is found which cures, and dismisses from all clinical labors the painful burdens of doubt and guessing, inhering in all efforts at curing not so directed?

If this be the true view of the case, then it is evident that a second reason for the delay of the needed "reform" in the education of those who are to be our healers is found in what seems to be a fundamental mistake as to just where "reform" is to begin; and a mistake here is failure, and a total failure, as to the result. The beginning of "reform" must be with the *teacher* and not the *pupil*. The reverse of this seems to have been the idea of those who have recommended, as a remedy for the acknowledged need of improvement, to add a year to the time of pupilage, with a third course of lectures to the two previously required, before examination and graduation could be granted to the pupil, thus beginning at the wrong end of the problem. More time? This may be well enough, and indeed not unfrequently very desirable. But more lectures? This may be a remedy of doubtful value; or, indeed, it may be no

* In an "Annual Announcement" of one of our colleges, just received, we find set apart to teach the knowledge which characterizes our school—that of materia medica and therapeutics—five professors, two lecturers, and three clinical assistants (whatever these may be); and to teach other branches of knowledge, aids to our therapeutics, twenty-eight professors, assistants, lecturers, demonstrators, etc. It would seem that here has been an attempt to remedy the felt deficiency by a multiplication of the number of teachers. In this attempt there would seem to be light on the ideas of this school as to what is required in a medical education—ten teachers of all grades to instruct in the essentials of the education and twenty-eight to teach cognate branches—a little like placing a cone on its apex.

remedy at all for this greatly needed "reform" in our educational endeavors and experiences. How is this *third* course of lectures—which is likely to be virtually a repetition of the two previous ones, which were mainly given to teachings of *those aids* to the science of therapeutics—to remedy the previous neglect of this most important of all sciences to the practical healer ?* The third course may make the pupil more thoroughly master of a knowledge of these aids, but not better acquainted with that more important science, to which these are but aids, if the neglect of this, so characteristic of the teachings of the two previous terms, is to be continued. Neither more time nor more lectures of the same character are any cure for this neglect, nor is any other expedient which begins and ends with the pupil. The "reform" can only begin and progress with the teacher.

If the education received by our graduated pupils in medicine is defective, the defect is to be charged to the teacher—neglect of duty from a lack of care or knowledge on his part—or to the pupil—want of capacity or industry on his part. In a given case both these causes may have been active in producing the sad result. But until the neglect of the teacher, now almost, if not quite, universal in our schools, to teach, and teach thoroughly, the true philosophy of our fundamental law and its necessary corollaries, is amended, there can be no improvement in the education they impart to young men who resort to their class-rooms that they may there acquire the knowledge necessary to constitute them expert healers. The very knowledge they came for has been withheld from them by the negligence or incompetence of those whose duty it was to teach the philosophy as well as the practice of our science of therapeutics. We hazard little, we believe, if we assert that this neglect is nearly, if not quite, universal in our homœopathic medical colleges. With other colleges we have no concern. We believe truth will sustain this charge of neglect which we make. We believe this because, in the first place, we have failed to recognize evidence of a knowledge of this philosophy in any conversation we have had

---

* In the "Announcement" already mentioned, an arrangement has been made to avoid the *repetition* here spoken of, by giving the student instruction in different branches of science in successive lecture terms. This is no doubt an improvement on the earlier method, which crowded all together, so that the pupil, at the end, was rather wearied than instructed. But it does not appear in this arrangement that there has been brought into it any plan or man to teach more or more thoroughly the fundamental and essential philosophy of our materia medica and therapeutics, and whatever of change or improvement which leaves this radical defect unprovided for, leaves the education of the pupil inadequate to the discharge of his prospective duties.

or heard with recent graduates, and when such have been met in assemblages of doctors, they have been silent as to all relating to this philosophy. They have seemed to have no inkling of an idea that there was any such philosophy in the world. They had come out from their colleges possessed of all they have had to impart, and in it all there has been found no apology even for this philosophy, so conspicuous by its absence from their ideas of needed medical knowledge. In the second place, we believe this because, when talking with students from two of our colleges, where, as we are informed, the largest classes are gathered, and which, we believe, as compared with the whole number of such colleges, are best furnished with talent in their teachers, who are as earnest and faithful as others, in their endeavors to instruct their classes, yet these students, each more than commonly endowed with general intelligence, and having just closed a lecture term, when asked as to whether they had been taught this philosophy, declared they had never heard of any such thing, and their conversation confirmed the impression of their utter ignorance of it. They had neither of them heard the *Organon* mentioned, from the beginning to the end of their lecture term, though in this is the clearest expression of that philosophy, so indispensable to a scientific practice of our therapeutics, and yet in pretended teaching of the young the knowledge they needed to constitute them expert healers, this indispensable element, had been almost wholly left out. Are we then in the wrong when we affirm that any reform in our elementary educational processes must begin in a reform of our teachers if there is to be realized a reform of any real value. And that this is to be carried into reforming them of their negligence, indolence, or ignorance, of each or all of these, where present, till a knowledge of the therapeutics they profess to teach shall be given to their pupils in all faithfulness and fullness, both in its science and practice.

If this be the true view of the case, what are we to think of the wisdom, or want of it, which thinks it has found a remedy for this fundamental defect in the body of our *teachers*, in extending the time in which *pupils* shall be exposed to the old, repeated short-comings which have all along left graduated pupils so nearly destitute of a knowledge which alone can constitute a basis of a scientific and successful practice of our divinely given system of therapeutics? If the result of this plan of relieving the teachers from the odium of a burden so justly belonging to them, by this proposed misuse of the pupil's time and means, were less important and less sad, this result, which we have heard boasted of as a great advance in educational experience by these teachers themselves, would at once relegate itself to the category of plans and resorts the most absurd. And the singular part of it all is the apparent want of consciousness on the part of these teachers that there is anything at all absurd in it. They do not seem to see that *quantity* of *time* can be no remedy for *defective quality* of *teaching*. Though it would appear, on the face of the suggestion, that no greater absurdity can possibly be inaugurated. If the idea has originated with these teachers, as seems to be the fact, is not its promulgation evidence of a blindness on their part as to the true nature of the needed reform rather discouraging as to any hope of this coming from any effort on their part to supply our great need? How can they be expected to teach the philosophy and practice of our therapeutics when the whole basic philosophy on which this science rests is so wholly absent from their own minds? It is unquestionably true that just here is the great defect in the education given to our young men who are sent out to treat the sicknesses of mankind by these institutions which are trusted to furnish them with the knowledge needed for their life work. The very core and essence of this knowledge, that of the science of therapeutics, which alone can give them the power to cure, has been so almost entirely left out. Curing is but the practical application of this science for relief of the sick. And yet students but recently from the class-room, at the close of the term of two of our best colleges, have assured me they have heard no word of the philosophy of this science from the first to the last lecture of the course. The science of therapeutics is left out. This is the defect in our educational process which so greatly needs to be cured. This, it is not necessary to repeat, cannot be accomplished by an additional term of lectures to be characterized by this repeated neglect.

Nor is it by any endeavor to bring our colleges into the most perfect likeness possible to those of old-school physic, by teaching the sciences, which are but aids to our therapeutics, as these are there taught. These aids are presumably well enough taught in both. The difficulty with us is that that to which these sciences are but aids is not taught at all, or so imperfectly that the graduate is wholly unable to apply it in the duty of practical healing. The question then is, if we are to have reform in our medical educational processes, the defect being in the *teachers*, whether this shall be realized by a reform *in* these teachers or by reforming them *out*. This, or the old, defective, unsatisfactory process continued—which? Is there any middle ground or course from which relief can come? Nay, but the reform must be more knowledge with the teacher and greater diligence in imparting this to the pupil. Then, if the pupil be found defective, let him bear the disgrace.—P. P. WELLS, in *Hom. Jour. of Obstetrics, etc.*

